# Parents’ and clinicians’ views of an interactive booklet about respiratory tract infections in children: a qualitative process evaluation of the EQUIP randomised controlled trial

**DOI:** 10.1186/1471-2296-14-182

**Published:** 2013-12-01

**Authors:** Nick A Francis, Rhiannon Phillips, Fiona Wood, Kerry Hood, Sharon Simpson, Christopher C Butler

**Affiliations:** 1 Cochrane Institute of Primary Care and Public Health, School of Medicine, Cardiff University, Neuadd Meirionnydd, Heath Park, Cardiff CF14 4YS, UK; 2 South East Wales Trials Unit, Institute of Translation, Innovation, Methodology & Engagement, School of Medicine, Cardiff University, Neuadd Meirionnydd, Heath Park, Cardiff CF14 4YS, UK

**Keywords:** Respiratory tract infections, Child, Antibiotic, Consulting, Qualitative, Process evaluation

## Abstract

**Background:**

‘When should I worry?’ is an interactive booklet for parents of children presenting with respiratory tract infections (RTIs) in primary care and associated training for clinicians. A randomised controlled trial (the EQUIP study) demonstrated that this intervention reduced antibiotic prescribing and future consulting intentions. The aims of this qualitative process evaluation were to understand how acceptable the intervention was to clinicians and parents, how it was implemented, the mechanisms for any observed effects, and contextual factors that could have influenced its effects.

**Methods:**

Semi-structured interviews were conducted with 20 parents and 13 clinicians who participated in the trial. Interviews were audio-recorded and transcribed verbatim. Data were analysed using a framework approach, which involved five stages; familiarisation, development of a thematic framework, indexing, charting, and interpretation.

**Results:**

Most parents and clinicians reported that the ‘When should I worry’ interactive booklet (and online training for clinicians) was easy to use and valuable. Information on recognising signs of serious illness and the usual duration of illness were most valued. The interactive use of the booklet during consultations was considered to be important, but this did not always happen. Clinicians reported lack of time, lack of familiarity with using the booklet, and difficulty in modifying their treatment plan/style of consultation as barriers to use. Increased knowledge and confidence amongst clinicians and patients were seen as key components that contributed to the reductions in antibiotic prescribing and intention to consult seen in the trial. This was particularly pertinent in a context where decisions about the safe and appropriate management of childhood RTIs were viewed as complex and parents reported frequently receiving inconsistent messages.

**Conclusions:**

The ‘When should I worry’ booklet, which is effective in reducing antibiotic prescribing, has high acceptability for clinicians and parents, helps address gaps in knowledge, increases confidence, and provides a consistent message. However, it is not always implemented as intended. Plans for wider implementation of the intervention in health care settings would need to address clinician-related barriers to implementation.

**Trial registration:**

ISRCTN46104365

## Background

Symptoms of respiratory tract infections (RTIs) are the most common reason for children to consult, and be prescribed antibiotics, in primary care [1,2]. Around 20% of children who consult for a RTI will re-consult for the same illness episode [3,4]. Unnecessary prescribing of antibiotics can cause side effects such as diarrhoea and rashes [5] and can increase antibiotic resistance [6]. Prescribing antibiotics for RTIs can also ‘medicalise’ the illness and drive further consultation for similar symptoms [7,8]. Given that RTIs in children are common and usually self-limiting, education and support for parents and clinicians could be of benefit in reducing unnecessary antibiotic prescribing and consultation [1,9].

Systematic reviews of interventions to reduce antibiotic prescribing for RTIs in children have indicated that a multi-faceted approach is likely to be required, where interventions occur on more than one level (e.g. clinicians, children, parents, and/or members of the public) [10,11]. Interventions targeting both parents and clinicians during consultations are most effective in reducing antibiotic prescribing for childhood RTIs, while passive educational strategies (such as waiting room educational materials) do not appear to be beneficial [11]. The widespread implementation of effective interventions to improve antibiotic stewardship for childhood RTIs in health care settings requires an understanding of the context in which an intervention was evaluated and how it could be adapted to other contexts [12]. Understanding the potential barriers to adoption of the intervention and how these might be overcome can be as important as the nature of the intervention itself [12]. Process evaluations are an important part of evaluating complex interventions, providing a better understanding of the implementation and receipt of intervention and the context in which they are delivered, which can help in the interpretation of outcome results [13].

The underlying reasons for inappropriate antibiotic use for respiratory infections, including aspects of the physician/patient interaction, physician characteristics (duration in practice, involvement in teaching, caseload), and uncertainty in identifying potential serious infection, have been studied extensively [14]. However, there is a sparsity of process evaluation of complex interventions to improve antibiotic stewardship in primary care. A qualitative evaluation of the Stemming the Tide of Antibiotic Resistance (STAR) Educational Program, a blended learning program to improve antibiotic prescribing for whole practice populations, indicated that many clinicians acknowledged the importance of improving antibiotic stewardship and felt that they had increased awareness of antibiotic resistance, greater confidence in making decisions about prescribing, and a greater insight into patient expectations following training [15]. However, we have not identified any published process evaluations of interventions to reduce antibiotic prescribing for childhood RTIs in primary care.

The ‘When should I worry’ interactive booklet for parents and associated training for clinicians is a complex intervention designed to empower parents and address high levels of re-consulting and inappropriate antibiotic prescribing for RTIs in children in primary care [16]. The booklet provides parents with information about RTIs in children and was developed using a systematic multi-stage process involving consultation with parents and clinicians [16]. The training was developed using a context bound communication skills training approach [17], and encourages clinicians to communicate more effectively, including asking about concerns and expectations and to use the booklet as a tool to facilitate discussion within the consultation [18]. Since the booklet and training were developed, the Behaviour Change Wheel (BCW) framework has been proposed to help characterise behaviour change interventions [19]. Within the context of the BCW, the booklet and associated training focus on changing motivation and capability to appropriately manage childhood RTIs for both parents and clinicians through training and education [19].

We evaluated this intervention in a cluster randomised controlled trial (RCT), the Enhancing the Quality of Information-sharing in Primary Care (EQUIP) for childhood respiratory tract infections study [18,20]. Use of the interactive booklet was associated with an approximately two-thirds reduction in antibiotic prescribing compared to usual care and a statistically significant reduction in future consulting intentions with no discernible reduction in parental satisfaction [18]. Furthermore, there were no statistically significant differences in parental reassurance, enablement, or re-consulting for the same illness within the two week follow up period [18].

In the current study, we carried out a qualitative process evaluation with the aim of understanding how acceptable the intervention was to clinicians and parents, how it was implemented, the mechanisms for any observed effects, and contextual factors that could have influenced its effects.

## Method

A qualitative process evaluation was carried out, using semi-structured interviews to explore parents’ and clinicians’ views of the intervention. Ethical approval for this study was provided by the South East Wales Research Ethics Committee and written informed consent was obtained from all participants.

### Setting and participants

In the EQUIP study, a RCT was carried out with 558 children (aged 6 months to 14 years) presenting to primary care with an acute respiratory tract infection (7 days or less) in 61 general practices in England and Wales [18]. Exclusion criteria were: children with suspected pneumonia, asthma or a serious concomitant illness, or needing immediate hospital admission.

Parents in the intervention arm of the trial who had been recruited after August 2007 (n = 93) were invited to participate in the qualitative interviews by letter during the last few of the study. Of these, 20 parents completed interviews, giving a response rate of 21.5%. Respondents were telephoned in order to conduct a telephone interview or arrange a suitable time to conduct a telephone interview. A maximal variation sampling framework was used to capture a cross-section of views according to whether:

1. Their child had been prescribed antibiotics at the index consultation.

2. The child had re-consulted within the two-week follow-up period.

All participating clinicians who were in a practice randomised to use of the intervention and who had recruited one or more patients into the trial (n = 51) were sent a letter at the end of participant recruitment inviting them to take part in a telephone interview. Thirteen of these participated in interviews (response rate 25.5%). Respondents were telephoned to conduct the interview or arrange a suitable time for conducting the interview. A maximal variation sampling framework was used to capture a cross-section of views according to whether:

1. The clinician was in a practice with an above or below average antibiotic prescribing rate.

2. The clinician had more or less experience in using the intervention (i.e. was a higher or lower recruiter than the average for practices included in the study, ≤6 participants or >6 participants recruited).

Average prescribing rates were estimated from 2005/2006 data from Health Solutions Wales and the Prescribing Pricing Authority in England. The rates were 700 items per 1000 patients in Wales and 617.8 items per 1000 patients in England. We aimed to recruit equal number of participants in each of the four cells of the sampling frameworks for both groups, but due to difficulties with recruitment, participants were recruited to other cells where we were unable to achieve this.

### Intervention

Clinicians in the intervention group were asked to use an interactive booklet, ‘When should I worry’, during consultations with recruited patients with RTIs and their parent and to provide it as a take home resource. The eight-page booklet contained information on symptoms of RTIs, usual course of illness, antibiotics, self-management, and when to seek help. The booklet was developed through a multistage process [16]. Clinicians were provided with online training describing the content and aims of the booklet, encouraging its use within consultations, and promoting good communication (exploring parents’ concerns, expectations, and discussing prognosis/treatment). The training included information and videos providing examples of tasks to be completed during consultations, and took approximately 30 minutes to complete. Both the booklet and training are available online at http://www.whenshouldiworry.com [accessed 24.10.13]. Clinicians in the control group conducted consultations as usual [20].

### Procedure

Parent interviews were carried out between one and four months from the index consultation to ensure this did not interfere with the primary short-term trial outcomes, but was early enough for a reasonable level of recall. Semi-structured interviews were guided by use of a topic guide, to provide a focus on relevant issues but without constraining interviews to pre-determined topics and thus allowing flexibility to explore salient emerging themes. The clinician interviews centred around the following topics: general impressions of taking part in the study, impressions of the booklet, how the booklet was used, views on the online training, views on whether the intervention had changed knowledge, beliefs or behaviour with regard to managing childhood RTIs, and more general views on the way in which childhood RTIs are managed. Parent interviews began by asking parents to talk about their child’s illness and what had prompted their visit to the surgery. The topic guide then moved on to general impressions of (and satisfaction with) the consultation, views on the booklet, how the booklet was used, whether the consultation/booklet had changed their beliefs, knowledge or behaviour with regard to managing childhood RTIs, and general views on the management of childhood RTIs and use of written information. It was anticipated that interviews would take 20–30 minutes to complete. Interviews were carried out by two researchers by telephone and were digitally audio-recorded.

### Analysis

Interviews were transcribed verbatim and checked for accuracy. Framework analysis was used, which is a systematic approach to qualitative analysis that allows for comparisons between and within cases, sharing and discussion of data, and allows for clear linking/access from developed themes to original data [21]. Framework analysis is particularly useful in the context of this study, where there were a number of clear research aims that guided the questions (i.e. investigating acceptability, how the intervention was implemented, and contextual factors), while allowing new themes to emerge from the data. Analysis involved five stages: familiarisation, development of a thematic framework, indexing, charting, and interpretation [22]. A summary of the analysis process applied in the current study is provided in Table 1.

**Table 1:** Summary of the qualitative framework analysis process applied in this study

**Stage**	**Process**
Stage 1: Familiarisation	Familiarisation with the data was first achieved by reading through all transcripts.
Stage 2: Framework development	A thematic framework was developed based on the main research questions and the main themes arising from the data. This is an index of categories or themes that is used to classify the data, and is usually arranged hierarchically. The initial coding framework was modified a number of times following discussions with the research team and during the coding process.
Stage 3: Indexing	Thematic codes were applied to all of the data which allowed data to be sorted, organised, and grouped.
Stage 4: Charting	Data coded by theme were retrieved and summarised in a chart. Each cell then contained a summary of how that theme applied to that participant and / or an indicative quotation from that transcript.
Stage 5: Interpretation	The final stage involved interpreting the data by drawing inferences and pulling together relevant themes.

Two parent interviews and one clinician interview were dual-coded to allow for an assessment of coding validity. Members of the research team with expertise in qualitative analysis (NF, FW, SS) met regularly during the analysis process to discuss the development of the coding framework, validity, and interpretation of the findings. This group assessed the level of agreement between coders, which was found to be good, with very little disagreement on the themes specified in the coding framework. Discrepancies were resolved through discussion. The research team also made an assessment of whether data saturation had been achieved, i.e. whether the point was reached when no major new themes were emerging from the data as analysis progressed. Saturation was deemed to have been achieved in both the clinician and parent interviews. The NVivo 8 was used to assist coding and data management.

## Results

Thirteen clinicians and 20 parents took part in the interviews. Characteristics of participating parents and clinicians are shown in Table 2.

**Table 2:** EQUIP qualitative process evaluation participants’ characteristics

**Parents**			
		Antibiotics received	
		Yes	No
Re-consulted for the same episode of illness	Yes	5	1
	No	5	9
**Clinicians**			
		Number of participants recruited by clinician	
		High, ≥ 6	Low, < 6
Practice antibiotic prescribing rate	Above average	2	3
	Below average	3	5

### Acceptability

#### Satisfaction with the ‘When should I worry’ booklet

Positive comments about the booklet were made by the majority of parents, describing it as ‘good’, ‘useful’ and ‘helpful’. However, some parents felt the information was ‘pretty obvious’ or they ‘knew most of the stuff in it’. There was also a high level of consensus amongst clinicians in reporting generally positive impressions of the booklet, describing it as ‘useful’, ‘clear’, ‘patient friendly’, and ‘well designed’.

“…it was nice to have such a friendly, such a patient friendly information booklet to give to parents of young children, I thought that was really useful”

[Clinician 159, below average prescribing practice, higher recruiter]

However, one clinician felt the booklet may be a bit too ‘erudite’ for some patients. Advice about recognising signs of serious illness and the normal duration of symptoms (including the graphical representations) were most frequently mentioned as useful parts of the booklet by both clinicians and parents.

#### Parental satisfaction with the consultation

When describing their satisfaction with the consultation, most parents talked about the manner of the clinician, and the thoroughness of the examination their child received. Many talked about feeling reassured during consultations;

“*I was really pleased she checked [name] over very thoroughly. I thought it wasn’t, you know, listen to me and say ‘ohh yeah, well he’s got a cold you know he’s gonna get over it’. I mean she [the doctor] checked his ears and examined his chest and listened to his chest and was, you know, reassuring”*

[Parent 544, no antibiotic, no re-consultation, index consultation with doctor]

Several parents recalled being specifically asked about their concerns, but very few discussed being asked about their expectations. However, parents didn’t typically feel that being asked about their expectations would have been helpful. A small minority of parents were not entirely satisfied with their consultation. For example, a parent described her dissatisfaction in that the clinician dedicated more time to enrolling her child into the study than dealing with the illness. However, after reflecting on the consultation at home she felt less dissatisfied:

“I have to say that when I came out I kind of felt fobbed off … but it wasn’t until I got home I thought well actually I am relieved because he has checked his front and his back, he’s confirmed it’s a cold, so I know he doesn’t need any antibiotics.”

[Parent 556, no antibiotic, no re-consultation, doctor]

Another parent who reported dissatisfaction had strong beliefs about the need for antibiotics, had expected a more thorough physical examination, and felt that she had not been given sufficient information.

#### Clinicians’ satisfaction with training

Clinician satisfaction with the training was somewhat variable. Several clinicians felt positive about the training, feeling that it was important and provided an opportunity to familiarise themselves with the content of the booklet.

“I thought the training was really excellent - the best bit of sort of introduction to study training that I’ve ever done because it forced you to interact with it”

[Clinician 98, below average prescribing practice, higher recruiter]

A clinician described a positive effect of training on their communication within consultations:

“Having had the training and using the booklet has helped me to consult and to improve my general consulting style in terms of eliciting the patient’s agenda and the patient’s priorities”

[Clinician 53, above average prescribing practice, lower recruiter]

Several clinicians were more neutral about the training, although few explicitly negative comments were made about it. Some clinicians felt that the training contained ‘obvious stuff’, while others remarked that it was useful in refreshing their knowledge within the context of a busy practice. For some clinicians, there was a gap between completing training and recruiting patients, which made it more difficult to recall the content. While a small number of clinicians reported some technical difficulties with the online training (e.g. not having working sound on their computer, difficulty logging on), the majority of clinicians were satisfied with the method of delivery of the training. None of the clinicians stated that they would have preferred face-to-face instead of web-based training.

### Implementation

Parent and clinician reports indicated that there was considerable variation in how the booklet had been used during consultations. While some parents reported that the booklet had been discussed with them during the consultation, for others it had not, and some had not received a booklet at all. In line with this, some clinicians reported using the booklet with every participating patient during consultations, while others used the booklet with only some participants or only partially used the booklet as instructed. There was a general consensus for both parents and clinicians that using the booklet interactively during consultations was important;

“I think if you just give it at the end, it wouldn’t carry much weight to be honest.”

[Clinician 98, below average prescribing practice, lower recruiter]

“I think it [being given a booklet] can seem like ‘oh well, here’s a booklet just read about it’, but you know, with sort of being shown the relevant parts it seems like they’re [clinician] taking more notice - they’ve listened to you and they’re trying to reassure you more”

[Parent 544, no antibiotic, no re-consultation, index consultation with doctor]

Many parents reported also reading the booklet at home following the consultation and several had kept the booklet for further reference. A small minority of parents expressed a preference to read through the booklet in their own time and one clinician felt that the information contained in the booklet was more important than the discussion of it.

Nonetheless, interactive use of the booklet during consultations did not appear to be consistently happening in practice. The main barriers to its reported use by clinicians were time, familiarity with use of the booklet during consultations, and discordance between the clinician’s treatment plan/style of consultation and the booklet’s messages. Additional time taken during consultations was a salient theme for clinicians. However, some clinicians considered this a reasonable cost for improving their patients’ understanding and ‘*made the consultation more constructive*’. Other less frequently reported barriers included feeling that the intervention might lead to use of a paternalistic (rather than shared decision-making) approach, and examples of slightly ’dysfunctional’ consultations where the communication style was perceived to have led to a loss of patient-focus and/or the usual quality of rapport was disrupted.

### Mechanisms and context

Clinicians emphasised the challenges involved in managing RTIs, including dealing with parents’ expectations, needs and desires, and the speed with which a child’s condition can change. Knowing how to respond when their child had an RTI was also challenging for parents, particularly in the context of often receiving inconsistent messages about the management of RTIs from healthcare professionals.

#### Antibiotic prescribing

Clinicians reported an increased understanding of antibiotic prescribing and awareness of parent perspectives as a result of the intervention;

“I’m more aware of the issues of antibiotic overuse and perhaps it’s led to me to think that well parents don’t always want antibiotics, they’re probably more likely to want reassurance in many of these cases.”

[Clinician 266, below average prescribing practice, lower recruiter]

For parents, feeling better informed about the role of antibiotics in managing RTIs and more confident in managing the illness without antibiotics having used the booklet was a salient theme;

“… with the ears. I think I was surprised at, they heal up on their own and you don’t need antibiotics. I just assumed that you need antibiotics every time you’re ill.”

[Parent 612, antibiotics, no re-consultation, index consultation with doctor]

Parents were generally receptive to messages that antibiotics were not always required. However, some parents reported confusion resulting from receiving conflicting messages from clinicians:

“*…the doctor was basically saying it might go away on its own anyway, but he felt that an antibiotic might or might not help. That didn’t really make sense….[ ]… I understand what they’re for and I understand the principle of not over-prescribing [antibiotics], but I think it’s a little confusing when they may have the same exactly the same symptoms one time and get them, and get antibiotics, and they feel that they need antibiotics on that occasion, and then the next time you go back and they’ve got identical symptoms you’re not given them.”*

[Parent 527, antibiotics, no re-consultation, doctor at index consultation]

Likewise, a number of clinicians talked about the damage done by inconsistent messages given to parents by clinicians, including actions (varying thresholds for prescribing antibiotics) and communication (conflicting advice). Use of the intervention may have encouraged clinicians to prescribe along more evidence-based lines:

“I suppose there were times when you have to try and overcome your own clinical prejudices to either go along with the booklet or not … the booklet was kind of construed, well, that mostly antibiotics are not helpful.”

[Clinician 173, above average prescribing practice, lower recruiter]

A clinician from a higher prescribing practice said that at the end of the study he felt like he ‘*explained more and prescribed less’*. Other clinicians talked about the booklet backing-up their advice or giving them more authority:

“…it’s not always easy when the expectation or perceived expectation is there for antibiotics, just in case. And it’s much easier to prescribe than not to prescribe. But if you’ve got something like that booklet then it kind of gives more backup or authority to reinforce the advice.”

[Clinician 15, below average prescribing practice, higher recruiter]

#### Re-consulting

There was considerable ambivalence around consulting the doctor from the parents’ perspective, with parents not wanting to be ‘a pain’, appear ‘paranoid’, ‘feel silly’ or ‘waste time’. Advice about recognising signs of serious illness and information about the usual duration of illness were most frequently mentioned as useful parts of the booklet, which was consistent with the high level of uncertainty parents reported around when they should consult with a doctor for a child’s RTI:

“The one thing that really stuck in my head is that these kind of infections last longer than you think….[]… [The doctor] was right because he said, and your booklet was right …a couple of days later and [name] was a different child… ”

[Parent 550, no antibiotic, no re-consultation, index consultation with doctor]

One parent reported that use of the booklet helped her obtain a timely consultation for a child with signs suggestive of serious illness;

“… it’s the fear of being a complete hypochondriac, and I thought ‘oh, let’s have a look at this booklet and see what it says’. And I read on a section you know, you should take back to your doctor if the child has very cold limbs and you know his hot body, and what have you, you should contact the doctor. So I did this and … she said come I’ll see him. And [the nurse] said you know, it just wasn’t the child she’d seen the day before. And his sats were low, his sats were 89”.

[Parent 594, antibiotic, re-consulted, index consultation with nurse]

For clinicians, similar themes of increased understanding of the natural history of RTIs and recognition of signs of serious illness emerged;

“Understanding the duration of symptoms a bit better than I did at the outset … you know, that mild symptoms can go on longer.”

[Clinician 173, above average prescribing practice, lower recruiter]

“I think I can more usually describe the signs of possible serious illness.”

[Clinician 184, below average prescribing practice, higher recruiter]

Some parents reported re-consulting primarily because they had been asked to by their clinician. Clinicians’ views also indicated that anxiety about not prescribing antibiotics may have increased re-consultations in some cases. For example, a clinician who had decreased his antibiotic prescribing reported that he tended to ask patients to return for follow-up more frequently to reassure himself they were recovering. Conversely, some parents felt that doctors could be quite dismissive;

“*Doctors do tend to sort of brush it off with it being a paranoid mother, which I can understand where they are coming from, but at the same time, you know, obviously she’s my daughter and I don’t see every other child with the same symptoms as well.”*

[Parent 518, no antibiotics, no re-consultation, index consultation with nurse]

One parent described feeling that they were being discouraged from seeing the doctor ‘in a nice way’ by the interactive booklet but also reported feeling more confident about differentiating between ’normal symptoms’ and signs of more serious illness. Parents often discussed the need for a thorough examination and reassurance from a health professional when their child was unwell, rather than a desire for specific treatment;

“*There’s just times when you know you need a bit of reassurance and a bit of peace of mind rather than actually going for a solution, you know*”

[Parent 555, no antibiotics, no re-consultation, index consultation with doctor]

## Discussion

### Summary of the main results

In this process evaluation we sought to understand the acceptability, implementation, and contextual factors influencing the effects of the ‘When should I worry’ booklet for parents and training for clinicians in its use. The majority of parents and clinicians found the booklet (and training for clinicians) easy to use and valuable, with the main criticism being that it was ‘obvious’ or ‘unnecessary’. Interactive use of the booklet was seen as important, but did not always happen. Lack of time, lack of familiarity with the booklet and difficulties in changing their approach to this condition were the main barriers to implementation described by clinicians. Increased knowledge and confidence amongst clinicians and parents were seen as key components that contributed to the reductions in antibiotic prescribing and intention to consult seen in the trial. This was particularly pertinent in light of the context in which this intervention was delivered, where the complex nature of decisions about managing childhood RTIs was highlighted and parents reported having previously had inconsistent messages about the management of RTIs.

### Comparison with existing literature

Satisfaction with consultations is a complex and multi-faceted issue, but the doctor-patient relationship, quality of communication and information, and clinical outcomes have been identified as important factors [23-25]. Individual patient preferences for styles of consultation vary, and not all patients want to participate in shared decision making when consulting for RTIs [26]. The findings of our study were consistent with this complex picture, indicating that while levels of satisfaction were generally good, individual parents’ and clinicians’ preferences need to be considered in consultations for childhood RTIs.

A multi-country qualitative study of views of clinicians in Europe of an internet-based program to reduce antibiotic prescribing indicated that in some countries people expected their doctor to make a decision or found certain communication strategies (such as being asked to sum up what they had learned at the end of a consultation) to be patronising, indicating that training for clinicians in communication around the management of RTIs needs to be sensitive to cultural and individual needs [27]. While the findings of our study highlighted the challenges of producing a ‘one size fits all’ training model for clinicians, providing training online was an efficient and convenient method of delivering training in use of the booklet which was generally acceptable, and none of the clinicians expressed a preference for face-to-face training as an alternative.

Clinician preferences need to be considered when implementing illness-focussed interventions such as the ‘When should I worry’ booklet, as these can affect adherence and uptake. Negative perceptions of the usefulness of illness-focussed interventions such as communication training for GPs who do not have direct experience of the intervention may act as a barrier to uptake and adoption [28]. Discrepancies between consultation length and communication style represented in training and the typical consultation for clinicians has previously been cited as a barrier to implementing an intervention to reduce antibiotic prescribing [27]. Similar barriers were reported by the clinicians in our study and ways of adapting the intervention to different contexts could potentially improve uptake and treatment fidelity. In the STAR study of an educational intervention to improve antibiotic prescribing practices in primary care, an emerging theme was that clinicians felt that there was additional time spent communicating with patients during the index consultation following training, but this had a positive trade-off in terms of reducing future consultation [15]. Although some of the clinicians reported feeling their consultations were more constructive when using the booklet, saving time on future consultations did not emerge as a theme in the current study, which was consistent with the lack of effect found in the quantitative data on re-consulting behaviour. Engaging with clinicians and ensuring that the benefits of investing time in these consultations in terms of antibiotic stewardship and improved knowledge of managing RTIs is likely to be vital in encouraging wider adoption.

Parents typically reported being receptive to the messages conveyed about antibiotics in the booklet, but both clinicians and parents highlighted the confusion caused by inconsistent messages about antibiotics and antibiotic prescribing behaviour. The majority of patients are aware that over-prescription of antibiotics contributes to antibiotic resistance [29]. In line with this, the findings of the current study suggested that parents were generally accepting of the message that antibiotics are not required for many cases of RTIs in children.

Perceived patient demand can have a significant influence on general practitioners’ prescribing behaviour for RTIs [30]. General practitioners may over-estimate expectations of patients for antibiotics during consultations [9]. The clinicians in our study reported a number of influences on their prescribing behaviour, including a tendency to explain more, becoming more aware of their tendency to over-estimate parents’ expectations for antibiotics, prescribing more along evidence-based guidelines, and using the booklet to back up their advice to parents regarding antibiotics. Increased awareness of antibiotic resistance, greater confidence in making decisions about prescribing, and a greater insight into patient expectations following training, were also noted in the STAR study [15].

The drivers for consultation for childhood RTIs in primary care are complex, and include the presence of prolonged or unusual symptoms, fever, poorer general health, unsuccessful self-management attempts, worry, need for reassurance, cues from others, parents’ willingness to tolerate somatic symptoms in their child, and parental mental health [1,16,31-33]. During the development of the ‘When should I worry?’ booklet parents expressed concern that the booklet should not deter parents from consulting if they are worried, and the booklet was designed with this in mind [16]. Problems for parents in assessing severity, challenging clinical authority, and worries about appearing ‘neurotic’, as well as access to healthcare and quality of consultations, can influence decisions to seek help for more serious RTIs [34]. From a clinician’s perspective, fears and uncertainty around diagnostic accuracy and the potential for missing serious illness have also been reported [16]. This highlights the challenge for both parents and clinicians in making safe, accurate and appropriate decisions about when to consult for RTIs. The uncertainty reported by parents in our study in relation to consulting was consistent with this complex picture of consulting behaviour. Furthermore, our findings indicated that in some cases clinicians shared some of the parents’ anxieties about safely managing childhood RTIs and had asked parents to re-consult. The extent of this practice is unknown, but if widespread this could have had an impact on the outcome of this study.

### Strengths and limitations

Qualitative data were systematically collected and analysed using a rigorous and well-established approach, providing an in-depth insight into parent and clinician views. We were unable to obtain equal numbers in each cell of our sampling frameworks due to difficulties with recruitment. Parents who did not receive antibiotics but did re-consult in particular are likely to have been under-represented. However, a range of views were obtained and data saturation was achieved. The sample was largely self-selecting and this should be considered in generalising from the findings.

## Conclusions

The ‘When should I worry’ booklet, which is effective in reducing antibiotic prescribing [18], has high acceptability for clinicians and parents, and helps improve knowledge and confidence and provide a consistent message about the management of RTIs in children. However, it is not always use interactively in the consultation as intended. Plans for wider implementation of the intervention in health care settings would need to address clinician-related barriers to implementation and acknowledge the complex drivers for consultation (focusing on enabling safe and appropriate judgements about when to consult).

## Abbreviations

RTI: Respiratory tract infection; RCT: Randomised controlled trial; EQUIP: Enhancing the quality of information-sharing in primary care for childhood respiratory tract infections

## Competing interests

The authors declare that they have no competing interests.

## Authors’ contributions

NF led the design, data collection, analysis and interpretation of these data and reporting on findings. NF and RP produced the first draft of this manuscript. RP and FW contributed to the qualitative analysis and interpretation. KH, CB and SS contributed to the overall design of the trial and process evaluation. All authors have been involved in critically revising this manuscript and have approved the final version.

## Authors’ information

NF is a Senior Clinical Research Fellow and a practising GP. He is the theme leader for Applied Infections research at the Cochrane Institute of Primary Care and Public Health at Cardiff University. RP is a Health Psychologist with expertise in qualitative process evaluation of complex interventions and child health. FW is a Medical Sociologist with a special interest in health care communication. KH has expertise in developing and evaluating complex interventions and is the director of the South East Wales Trials Unit. SS is a Behavioural Scientist with expertise in behaviour change and methodological expertise in developing and evaluating complex interventions. She is Associate Director of the South East Wales Trials Unit and leads the behaviour change theme. CB is the Director of the Institute of Primary Care and Public Health at Cardiff University and the Wales School of Primary Care Research. His main research interests are common infections and the appropriate use of antibiotics, and health behaviour change.

## Pre-publication history

The pre-publication history for this paper can be accessed here:

http://www.biomedcentral.com/1471-2296/14/182/prepub
